# Baicalein Inhibits Cerebral Ischemia-Reperfusion Injury through SIRT6-Mediated FOXA2 Deacetylation to Promote SLC7A11 Expression

**DOI:** 10.1523/ENEURO.0174-24.2024

**Published:** 2024-10-04

**Authors:** Cuini Fang, Xirong Liu, Fuxiu Zhang, Tao Song

**Affiliations:** Hunan Provincial People’s Hospital (The First Affiliated Hospital of Hunan Normal University), Changsha, Hunan Province 410000, People’s Republic of China

**Keywords:** baicalein, cerebral ischemia-reperfusion injury, FOXA2, ferroptosis, SIRT6, SLC7A11

## Abstract

Ischemic stroke (IS) poses a serious threat to patient survival. The inhibition of ferroptosis can effectively alleviate ischemia-reperfusion (I/R) injury, suggesting potential targets in the ferroptosis pathway for the treatment of IS. In this study, MCAO/R mice and OGD/R-induced HT22 cell were constructed. It was found that baicalein decreased ROS, MDA, and Fe^2+^ levels, upregulated GSH levels, and enhanced the expression of ferroptosis-related proteins (GPX4 and SLC7A11), downregulated the expression of proapoptotic proteins (Bax, cytochrome *c*, and cleaved caspase-3), and upregulated the expression of an antiapoptotic protein (Bcl-2), ameliorating cerebral I/R injury. In animal and cell models, Sirtuin6 (SIRT6) is downregulated, and Forkhead boxA2 (FOXA2) expression and acetylation levels are abnormally upregulated. SIRT6 inhibited FOXA2 expression and acetylation. Baicalein promoted FOXA2 deacetylation by upregulating SIRT6 expression. FOXA2 transcriptionally inhibits SLC7A11 expression. In conclusion, baicalein inhibited apoptosis and partially suppressed the role of ferroptosis to alleviate cerebral I/R injury via SIRT6-mediated FOXA2 deacetylation to promote SLC7A11 expression.

## Graphical abstract

**Figure:**
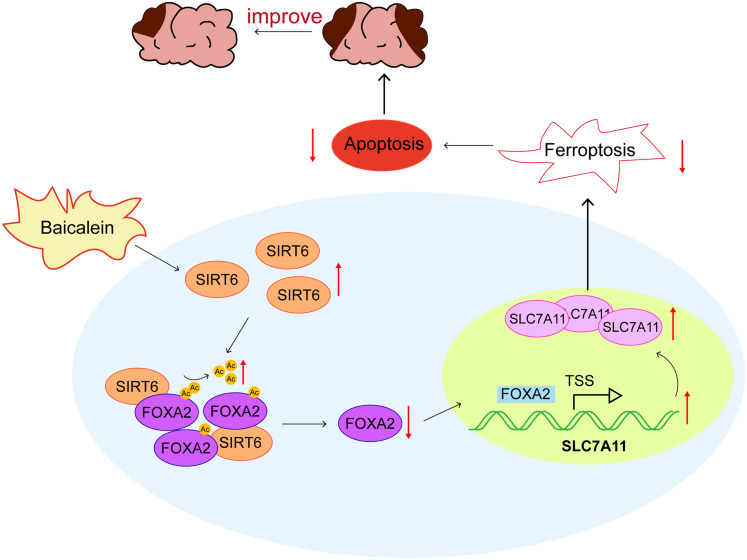
Visual Abstract

## Significance Statement

Ischemic stroke (IS) is a disease of the central nervous system with high mortality and morbidity rates. Currently, effective treatments for IS are limited. Therefore, it is urgent to develop novel treatment methods. Ferroptosis inhibitors and iron chelating agents can effectively alleviate IS neuronal damage, suggesting potential targets in the ferroptosis pathway for the IS treatment. This study confirmed, for the first time, that baicalein promoted FOXA2 deacetylation by upregulating SIRT6 expression, thereby inhibiting FOXA2 transcription, leading to the upregulation of SLC7A11 expression, inhibition of apoptosis, and partial suppression of the role of ferroptosis, thus inhibiting cell apoptosis and ultimately alleviating I/R injury in IS. Our study suggests that SIRT6/FOXA2 is a target of baicalein in IS therapy.

## Introduction

Ischemic stroke (IS) is a disease of the central nervous system with high mortality and morbidity (Meng et al., 2022). Vascular recanalization to restore blood supply is the main treatment measure for IS; however, it is easy to induce cerebral ischemia-reperfusion (I/R) injury, often accompanied by inflammation, ferroptosis, and oxidative stress, which is not conducive to the prognosis of patients (Campbell and Khatri, 2020). Currently, there are very few effective treatments for IS, and existing treatment methods have strict indications and certain risks, so there is an urgent need to develop novel treatments. Ferroptosis is a nonclassical form of programmed cell death characterized by iron-dependent lipid peroxidation (X. Chen et al., 2021b) and regulatory cell death caused by the accumulation of lipid peroxides and reactive oxygen species due to abnormal intracellular iron metabolism (Costa et al., 2023). Ferroptosis inhibitors and iron chelating agents can effectively alleviate neuronal damage during IS, suggesting potential targets for the treatment of IS (Zhan et al., 2023; Zhou et al., 2023).

Baicalein is a natural flavonoid with low toxicity that can reduce the risk of apoptosis, decrease MCAO/R mouse infarct volume, and alleviate neurological dysfunction in cerebral I/R injury (Ran et al., 2021). Yuan et al. found that baicalein possess a neuroprotective effect on I/R injury through NF-κB, LOX, and AMPK/Nrf2 (nuclear factor erythroid 2-related factor; Yuan et al., 2020). Additionally, baicalein is a natural ferroptosis inhibitor that not only effectively inhibits the formation of Fe^2+^ and decreases GSH consumption but also inhibits the degradation of GPX4, inhibits cell membrane lipid peroxidation, and prevents cell death, thus reversing cerebral I/R injury (M. Li et al., 2022). However, there are few reports on the regulation of ferroptosis by baicalein, and the specific mechanism requires further exploration. Baicalein inhibited ferroptosis by regulating SIRT1/p53 acetylation, thereby ameliorating polymyxin B-induced acute renal injury (Yu et al., 2023). Sirtuin6 (SIRT6) is a pluripotent lysine deacetylase belonging to the same family as SIRT1 (Chang et al., 2020; Liu et al., 2021). Recent studies have shown that SIRT6 regulates ferroptosis in gastric and pancreatic cancers (Cai et al., 2021; Gong et al., 2022). Hu et al. revealed that SIRT6 expression is decreased in brain endothelial cells stimulated by oxygen and glucose privation reperfusion (OGD/R) injury (Hu et al., 2015). Xiao et al. confirmed that exocrine signals produced by bone marrow mesenchymal stem cells can upregulate SIRT6 and ameliorate IS (Yuan et al., 2020). Therefore, we speculated that baicalein inhibits ferroptosis and alleviates I/R injury via SIRT6 regulation.

The cXc system encodes a key gene involved in the regulation of ferroptosis (Poltorack and Dixon, 2022). SLC7A11 is primarily expressed in the brain (W. Chen et al., 2021a). The inhibition of SLC7A11 expression reduces cystine uptake and GSH synthesis, leading to the weakening of antioxidant capacity and cell ferroptosis (Lei et al., 2021). Importantly, there are potential FOXA2 binding sites near the SLC7A11 promoter. As FOXA2 transcription can regulate the expression of its downstream target genes (Gao et al., 2022), we speculated that SIRT6-mediated FOXA2 deacetylation regulates SLC7A11 expression, thus regulating ferroptosis in IS.

This study aimed to explore whether baicalein regulated the SIRT6/FOXA2/SLC7A11 axis and inhibits ferroptosis, thereby inhibiting apoptosis and alleviating I/R injury in IS, providing a fresh perspective for the treatment of IS.

## Material and Methods

### Ethics approval and consent to participate

All animal experiments were completed under the regulations approved by Ethics Committee of Hunan Provincial People’s Hospital (The First Affiliated Hospital of Hunan Normal University).

### MCAO/R mice

C57BL/6 male mice (22–24 g, 6–8 weeks old; Weitong Lihua) were randomly divided into a sham group (*n* = 9) and the remaining 63 mice were used to construct the MCAO/R mouse model. MCAO/R mice were randomly divided into seven groups (*n* = 9/group): MCAO/R, MCAO/R + baicalein (100 mg/kg, the treatment time was 1, 4, and 7 d, respectively), MCAO/R + baicalein (treatment for 7 d) + sh-NC (5 × 10^8^ TU/ml, 2 μl) group, MCAO/R + baicalein (treatment for 7 d) + sh-SIRT6 (5 × 10^8^ TU/ml, 2 μl, GenePharma), and MCAO/R + baicalein (treatment for 7 d) + sh-SIRT6 + ferrostatin-1 (2 mg/kg, batch number: S724302) group. Mice were anesthetized with 50 mg/kg pentobarbital and subjected to MCAO. The common, internal, and external carotid arteries of the mice were segregated, and the thread infiltrated the internal carotid artery to obstruct the middle intracranial artery for 2 h and was then eliminated for blood reperfusion for 24 h. Mice with gait unsteadiness or forelimb immobility after surgery were used in this study, and mice without thread obstruction were used as the sham group. In the treatment group, the sh-SIRT6 or sh-NC plasmid was injected into the lateral ventricle of MCAO/R mice 24 h before perfusion (X. Y. Chen et al., 2021c). After model construction, baicalein or ferrostatin-1 was injected intraperitoneally, and the same amount of solvent was administered to the sham and vehicle groups (Wu et al., 2020; Zhu et al., 2022). All animal experiments were approved by the Ethics Committee of our hospital.

### Triphenyltetrazolium chloride staining

The forebrain along the coronal cut into slice thickness of 2 mm wafer, nurtured in 2% triphenyltetrazolium chloride (TTC) solution (Sigma-Aldrich) at 37°C for 30 min, and fixed with 4% paraformaldehyde overnight. The ImageJ (National Institutes of Health) analysis system was used to collect the cerebral infarction area and brain section area of the mice and calculate the cerebral infarction volume. Infarct area (%) = [contralateral hemisphere area − (ipsilateral hemisphere area − infarct area)] / contralateral hemisphere area × 100

### Neurological deficit score

Neurological deficit scores (NDSs) have been used to evaluate neural function in mice (Wang et al., 2023). The specific scores were as follows: a score of 0 indicated normal mouse activity and no obvious nerve function defect; 1 point, mild neurological dysfunction occurred in mice, and the front paw of the lesion could not be wholly extended; 2 points, the mice developed moderate neurological dysfunction and turned circles on the opposite side of the lesion; 3 points, the mice developed severe neurological dysfunction and fell to the opposite side of the lesion; 4 points, the mice had disturbances in consciousness and could not walk spontaneously; and 5 points, mice died.

### Cell culture

HT22 cells were purchased from Tongpai Biotechnology and cultured in DMEM supplemented with 10% fetal bovine serum (FBS; Invitrogen). HT22 cells were washed in glucose-free DMEM medium (Invitrogen), and then HT22 cells were incubated with an air flow of 1% O_2_, 5% CO_2_, and94% N_2_ at 37°C for 6 h. Subsequently, normal DMEM medium (Invitrogen) encompassing 4.5 g/L glucose and/or baicalein (10 μM) was incubated in a normal incubator for 24 h to construct the OGD/R model (M. Li et al., 2022).

### Cell transfection

Lentiviral vectors encoding short-hairpin SIRT6 (sh-SIRT6) and empty lentiviral vectors were constructed using GeneChem. pcDNA3.1-SIRT6 (Oe-SIRT6), pcDNA3.1-FOXA2 (Oe-FOXA2), and the corresponding negative control (NC) plasmids were obtained from GenePharma. Following the directions of Lipofectamine 3000 transfection reagent (Invitrogen), these plasmids were transfected for 48 h. Ferroptosis inhibitor ferrostatin-1 (1 µM, batch number: S724302), HDAC family inhibitors (TSA, 5 µM, batch number: S104509), and Sirtuin family inhibitors (NAM, 20 mM, batch number: S189901) were used to treat HT22 cells for 24 h, respectively.

### CCK-8 assay

The cells (6 × 10^3^ ml^−1^, 100 μl) were incubated into 96-well plates. After culturing for 24 h, each well was conflated with 10 μl of CCK-8 solution (Beyotime) for 4 h. The absorbance was measured using a Multiskan FC enzyme labeling instrument (Thermo Fisher Scientific) at a wavelength of 450 nm.

### Lipid ROS assay

Lipid ROS levels in HT22 cells were ascertained using C11-BODIPY (C10445; Invitrogen) according to the manufacturer's instructions. Briefly, treated cells were incubated with 10 mmol/L C11-BODIPY at 37°C and 5% CO_2_ for 30 min in the dark. After washing with phosphate-buffered saline (PBS), the fluorescence signal was detected using flow cytometry (excitation wavelength, 510 nm; emission wavelength, 590 nm).

### Flow cytometry analysis

Cells (5 × 10^4^ ml^−1^) were cultured in six-well plates with 2 ml of cell suspension per well and incubated for 48 h. The HT22 cells were then collected and washed. The supernatant was removed using centrifugation. Five microliters of Annexin V-FITC/PI (Absin) were added to the suspension under unilluminated conditions for 15 min. The fluorescence intensity was determined using flow cytometry (Agilent) immediately after the reaction was completed.

### MDA and GSH determination

MDA and GSH were identified using MDA (Beyotime) and GSH Assay Kits (Beyotime), respectively, according to the manufacturer's instructions. The optical density was evaluated using an automatic microplate reader (BioTek).

### Intracellular Fe^2+^ assay

The levels of Fe^2+^ and total iron were estimated using an iron assay kit (Dojindo) according to the manufacturer's instructions. The absorbance was measured at 593 nm using an automatic microplate reader (BioTek).

### Dual-luciferase reporter gene assay

The JASPAR database (https://jaspar.genereg.net/) was used to identify the binding sites of the FOXA2 transcription factor and SLC7A11 promoter. The wild-type (WT) and mutant (MUT) target sites at the SLC7A11 3′ UTR were amplified using PCR and cloned into a pmirGLO vector (Sangon Biotech) to construct the SLC7A11-WT and SLC7A11-MUT. The cells were cotransfected with the above vectors and Oe-FOXA2 or the corresponding control. After 48 h, the relative luciferase activity was determined using a dual-luciferase reporter assay (Promega).

### Chromatin immunoprecipitation assay

HT22 cells were lysed using RIPA lysis buffer (Beyotime) and 100 μl of lysate was incubated with RIPA lysis buffer encompassing magnetic beads conjugated to anti-FOXA2 or IgG. The proteins were then digested. The chromatin immunoprecipitation (ChIP) antibody was mixed overnight at 4°C, and DNA/protein precipitate was obtained. The DNA was isolated and purified for qPCR analysis.

### Western blot

The supernatant was collected, and the protein concentration in the supernatant was determined using the BCA method. An appropriate amount of protein was added to the sample buffer solution, boiled for 5 min, and subjected to sodium dodecyl sulfate polyacrylamide gel electrophoresis. After electrophoresis, the proteins were electrotransferred to a polyvinylidene diﬂuoride filter membrane and enclosed in 5% skim milk at ambient temperature for 1 h. Subsequently, the membranes were incubated with the appropriate primary and secondary antibodies. Finally, the bands were cultured with an ECL luminescence solution (Thermo Fisher Scientific) and transferred to a chemiluminescence imaging system for exposure development. The relative expression of each protein was ascertained using ImageJ (National Institute of Health). The following antibodies were used: GPX4 (ab125066, 1:1,000, Abcam), SIRT6 (ab191385, 1:2,000, Abcam), SLC7A11 (ab307601, 1:1000, Abcam), FOXA2 (ab108422, 1:1,000, Abcam), ACSL4 (ab155282, 1:10,000, Abcam), Bax (ab32503, 1:1,000, Abcam), Bcl-2 (ab182858, 1:2,000, Abcam), cytochrome *c* (ab133504, 1:5,000, Abcam), cleaved caspase-3 (ab214430, 1:5,000, Abcam), and β-actin (ab8245, 1 µg/ml, Abcam, as internal reference).

### Quantitative real-time PCR

Total RNA was extracted from tissues or cultured cells using the TRIzol reagent. The RNA concentration was measured using an ultraviolet-visible spectrophotometer (Thermo Fisher Scientific), and cDNA was synthesized using a one-step reverse transcription kit (Invitrogen). The reaction system and conditions were prepared according to the instructions for the Lipofectamine3000 kit (Invitrogen). β-Actin was used as internal parameters, and the expression of related factors was calculated using the 2^−ΔΔCt^ method. The primer sequences used were as follows: SIRT6, 5′-GTCAGAGACACGGTTGTGGG-3′ (Forward) and 5′-TCATCAGCGAGCATCAGGTC-3′ (Reverse); FOXA2, 5′-ATGCGTTACGTATTGCC-3′ (Forward) and 5′-GAACTAGGCTAGCTTAGC-3′ (Reverse); SLC7A11, 5′-CTATTTTACCACCATCAGTGCG-3′ (Forward) and 5′-ATCGGGACTGCTAATGAGAATT-3′ (Reverse); β-actin, 5′-CGTCCGTGACATCAAGGAGAAGC-3′ (Forward) and 5′- ACCGAGGAAGGAAGGCTGGAAG-3′ (Reverse).

### Statistical analysis

SPSS23.0 statistical software (IBM SPSS software) was used for the data analysis. The data were expressed as the mean ± standard deviation. One-way ANOVA was used to distinguish the differences among groups. Student's *t* test was used to discriminate between the two groups. All experiments were repeated three times. *p < 0.05* was expressed as statistically significant.

## Results

### Baicalein upregulated SIRT6 expression to inhibit apoptosis and partial role of ferroptosis, thereby alleviating I/R injury in mice

**Figure 1.**
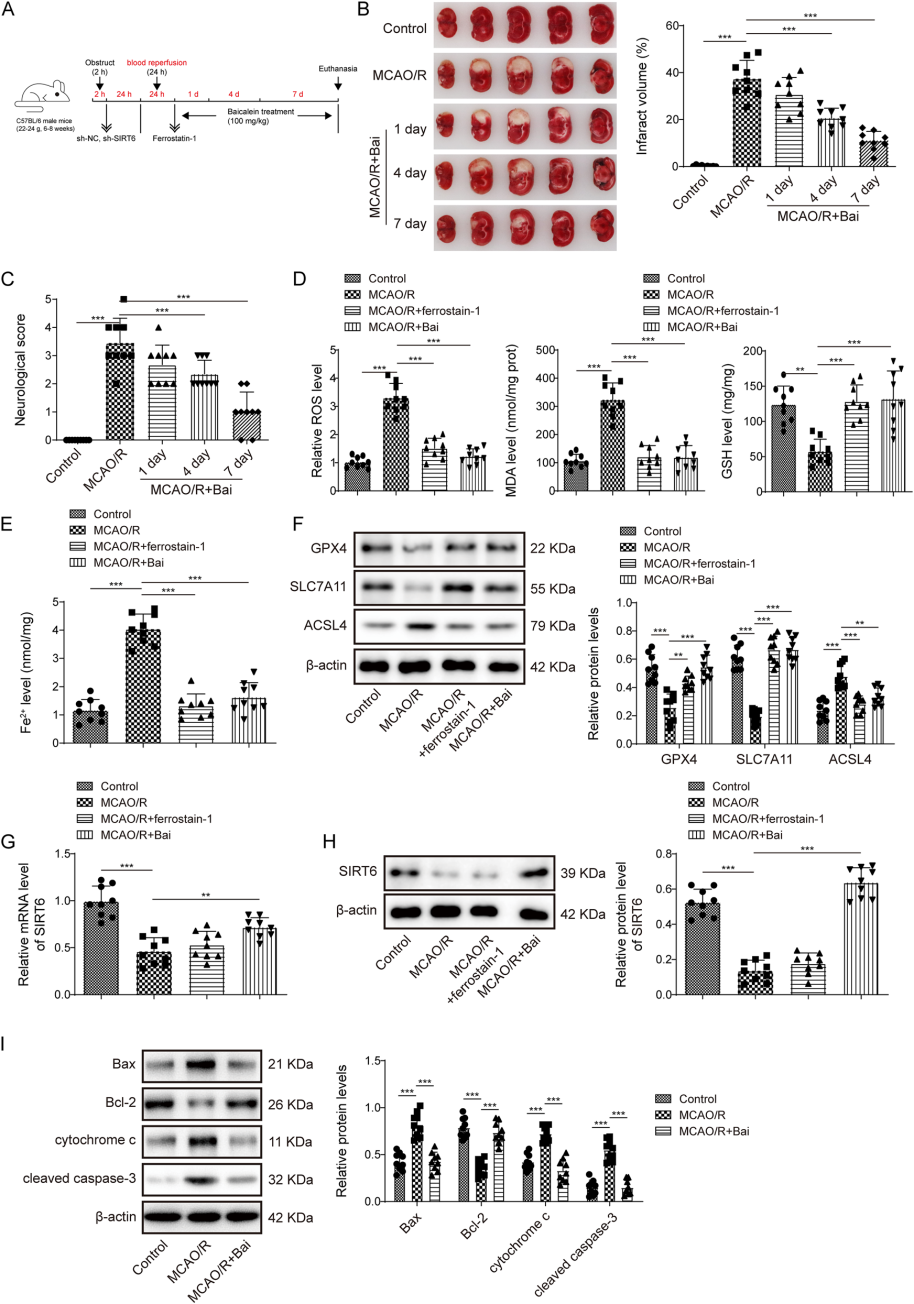
Baicalein upregulated SIRT6 expression to inhibit apoptosis and partial role of ferroptosis, thereby alleviating I/R injury in mice. MCAO/R mice were treated with baicalein. ***A***, Timeline for baicalein treatment. ***B***, The infarct size was measured using TTC staining. ***C***, Neurological deficit scores were used to evaluate the nerve injuries. ***D***, ***E***, The levels of MDA, GSH, Fe^2+^ were detected using kits. ***F***, Expression of GXP4, SLC7A11, and ACSL4 was determined using Western blotting. ***G***, ***H***, SIRT6 mRNA and protein expression were ascertained by qRT-PCR and Western blotting. ***I***, After 7 d of baicalein treatment, the expression of apoptosis-related proteins was ascertained by Western blotting. *n* = 9. *p < 0.05, **p < 0.01, ***p < 0.001.

Baicalein is believed to alleviate I/R injury (W. H. Li et al., 2020), but its specific mechanism has not been further confirmed. Therefore, we constructed an MCAO/R mice model and treated the MCAO/R mice with baicalein at different times (Fig. 1*A*). It was noted that the infarct size and neurological deficit score of MCAO/R mice increased, whereas the infarct size of baicalein-treated MCAO/R mice decreased gradually and the neurological deficit score decreased significantly (Fig. 1*B*,*C*). Notably, the effect of baicalein treatment was the most significant on Day 7. Therefore, baicalein treatment for 7 d was selected for follow-up experiments. Additionally, in the brain tissue of MCAO/R mice, the levels of MDA and ROS increased, and the level of GSH decreased (Fig. 1*D*). The results showed that in MCAO/R mice, Fe^2+^ levels were increased, ferroptosis-related protein (GPX4 and SLC7A11) expression was downregulated, and the expression of ACSL4 was upregulated (Fig. 1*E*,*F*). After treatment with a ferroptosis inhibitor (Ferrostain-1) or baicalein, all the above indices were reversed (Fig. 1*D–F*). Moreover, SIRT6 mRNA and protein expression was abnormally inhibited in MCAO/R mice. Baicalein increased SIRT6 expression, while Ferrostain-1 had a marginal effect on SIRT6 expression (Fig. 1*G*,*H*). Studies have shown that ferroptosis is closely related to apoptosis, apoptosis can be transformed into ferroptosis under certain conditions, and ferroptosis promotes the sensitivity of cells to apoptosis (Yi et al., 2020). The results revealed that the expression of proapoptotic proteins (Bax, cytochrome *c*, and cleaved caspase-3) was upregulated and the expression of antiapoptotic protein (Bcl-2) was downregulated in MCAO/R mice, and this improvement was ameliorated after treatment with baicalein (Fig. 1*I*). These results suggest that baicalein could alleviate nerve injury in MCAO/R mice brain tissue by inhibiting apoptosis and the partial role of ferroptosis, and this mechanism might be achieved by upregulating SIRT6 expression.

### Baicalein inhibited ferroptosis in OGD/R-induced HT22 cells by upregulating SIRT6 expression

**Figure 2.**
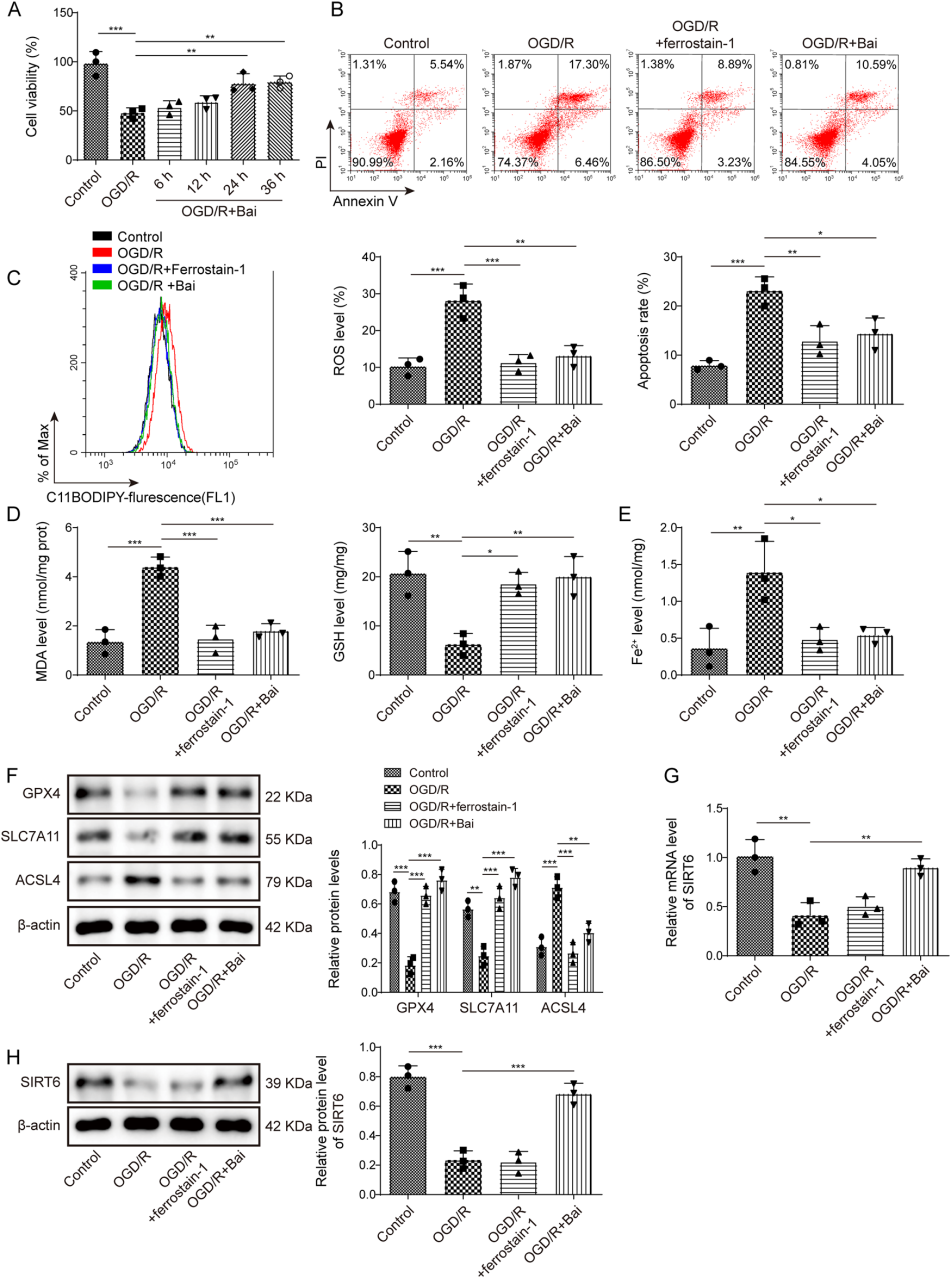
Baicalein inhibited ferroptosis in OGD/R-induced HT22 cells by upregulating SIRT6 expression. OGD/R-treated HT22 cells were treated with baicalein. ***A***, Cell viability was evaluated using a CCK-8 assay. ***B***, The cell death rate was determined by flow cytometry. ***C–E***, Lipid ROS, MDA, GSH, and Fe^2+^ levels were measured using kits. ***F***, GXP4, SLC7A11, and ACSL4 expression levels were determined using by western blotting. ***G***, ***H***, Expression of SIRT6 mRNA and protein was substantiated by qRT-PCR and Western blotting. Data from each study were tested three times. *p < 0.05, **p < 0.01, ***p < 0.001.

We then further explored the function of baicalein at the cellular level. In OGD/R-induced HT22 cells, HT22 cell viability decreased, and the cell death rate increased significantly. After treatment with baicalein, the viability of HT22 cells increased, and this effect was most significant 24 h after baicalein treatment (Fig. 2*A*). The rate of cell death decreased in OGD/R-induced HT22 cells after treatment with Ferrostain-1 or baicalein (Fig. 2*B*). In addition, in OGD/R-induced HT22 cells, the levels of lipid ROS and MDA were increased, and the level of GSH was decreased (Fig. 2*C*,*D*). Furthermore, Fe^2+^ level was increased, and the expressions of GPX4 and SLC7A11 were decreased in OGD/R-induced HT22 cells, while the expression of ACSL4 increased (Fig. 2*E*,*F*). Furthermore, the above indices were reversed in OGD/R-induced HT22 cells treated with Ferrostain-1 or baicalein (Fig. 2*C–F*). Finally, SIRT6 mRNA and protein expression were inhibited in OGD/R-induced HT22 cells. Baicalein effectively upregulated the expression of SIRT6, whereas Ferrostain-1 did not affect SIRT6 expression (Fig. 2*G*,*H*). These results indicate that baicalein inhibits ferroptosis in OGD/R-induced HT22 cells by upregulating SIRT6 expression.

### Baicalein alleviated OGD/R-induced HT22 cells injury through SIRT6-mediated partial role of ferroptosis

**Figure 3.**
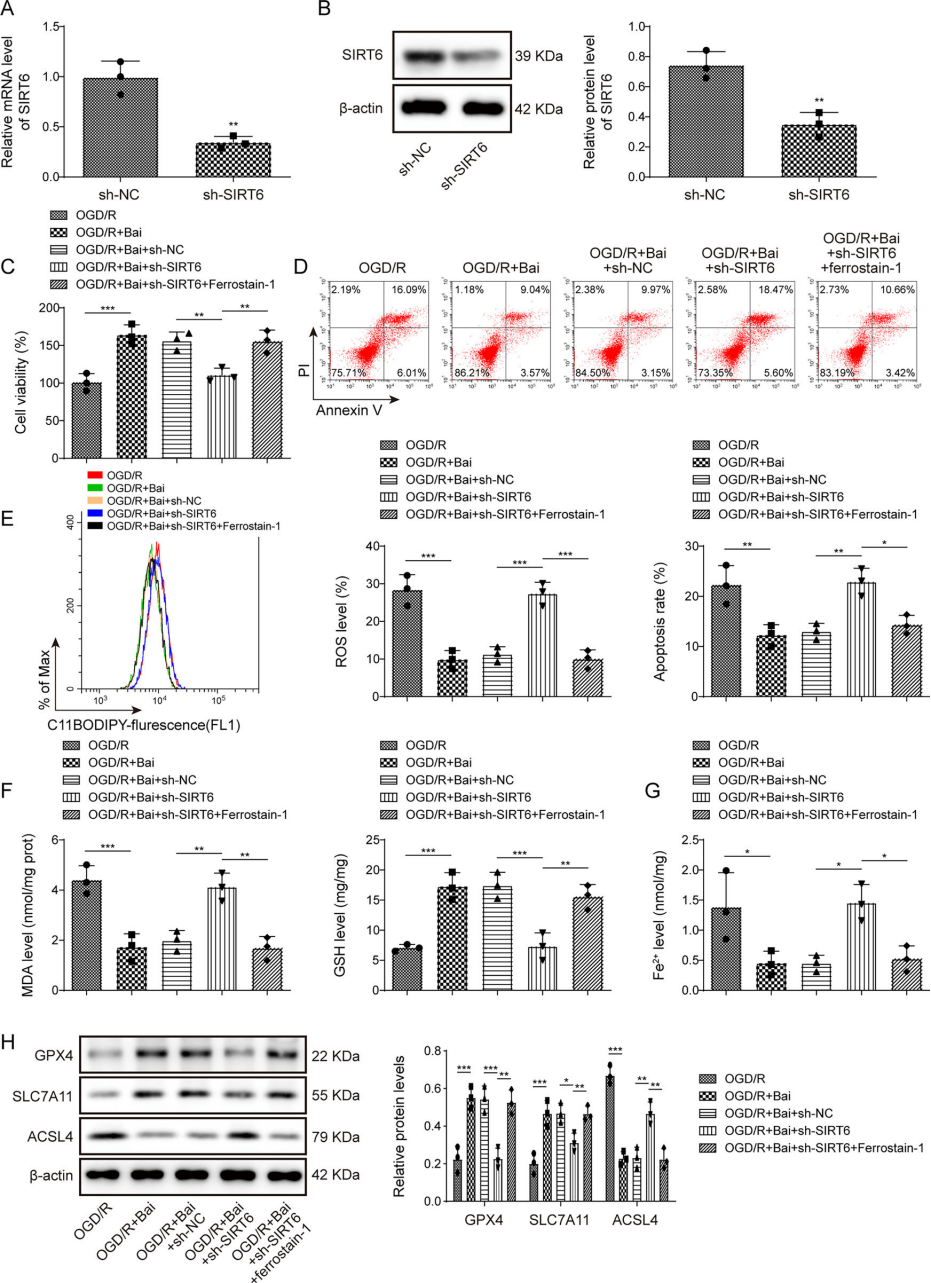
Baicalein alleviated OGD/R-induced HT22 cells injury through SIRT6-mediated partial role of ferroptosis. OGD/R-induced HT22 cells were treated with baicalein and transferred with sh-SIRT6. ***A***, ***B***, Expression of SIRT6 mRNA and protein was confirmed by qRT-PCR and Western blotting. ***C***, Cell viability was determined using the CCK-8 assay. ***D***, The cell death rate was ascertained using flow cytometry. ***E–G***, Lipid ROS, MDA, GSH, and Fe^2+^ levels were measured using kits. ***H***, Expression of GXP4, SLC7A11, and ACSL4 was determined using Western blotting. Data from each study were tested thrice. *p < 0.05, **p < 0.01, ***p < 0.001.

SIRT6 has also been identified as a regulator of I/R injury (Ruan et al., 2020). To explore this, we knocked down SIRT6 expression in HT22 cells by transfection with sh-SIRT6 (Fig. 3*A*,*B*). SIRT6 knockdown decreased the viability and aggravated the death of OGD/R-induced HT22 cells, thereby impairing the protective effect of baicalein (Fig. 3*C*,*D*). Meanwhile, Ferrostain-1 attenuated the inhibitory effect of SIRT6 knockdown (Fig. 3*C*,*D*). Furthermore, sh-SIRT6 upregulated lipid ROS and MDA levels and decreased GSH levels (Fig. 3*E*,*F*). Moreover, sh-SIRT6 increased Fe^2+^ levels, inhibited SLC7A11 and GPX4 expression, and promoted ACSL4 expression in baicalein-treated I/R cells (Fig. 3*G*,*H*). Interestingly, Ferrostain-1 attenuated the function of sh-SIRT6 (Fig. 3*G*,*H*). These results revealed that baicalein alleviated OGD/R-induced HT22 cell injury through a SIRT6-mediated partial role in ferroptosis.

### Baicalein promoted FOXA2 deacetylation by upregulating SIRT6

**Figure 4.**
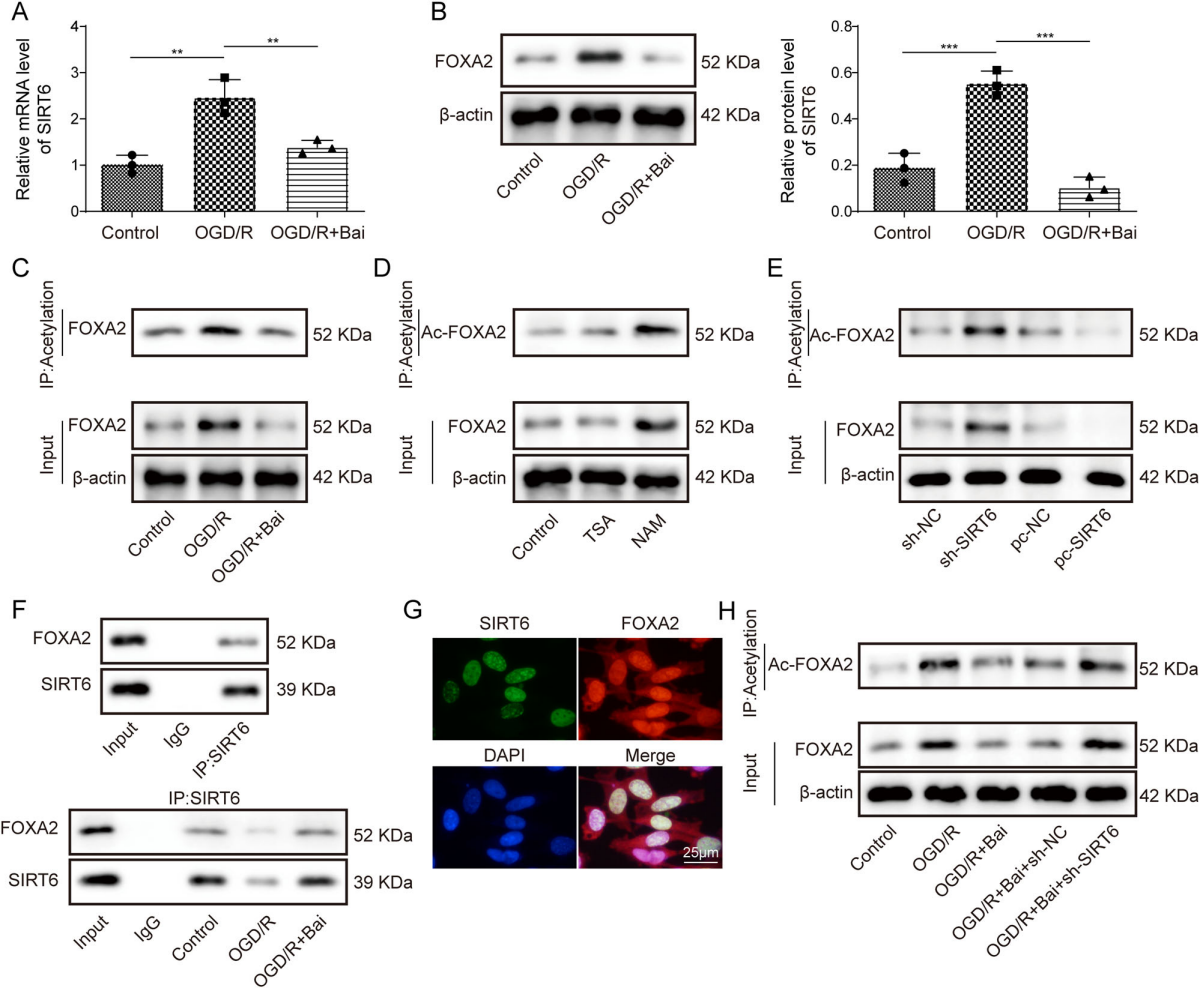
Baicalein promoted FOXA2 deacetylation by upregulating SIRT6. ***A***, ***B***, FOXA2 mRNA and protein expressions were substantiated by qRT-PCR assay and Western blotting. ***C–E***, FOXA2 expression and acetylation was substantiated by IP assay. ***F***, The regulatory relationship of FOXA2 with SLC7A11 was ascertained by Co-IP assay. ***G***, SIRT6 and FOXA2 were colocated in the nucleus. ***H***, FOXA2 expression and acetylation was ascertained by IP assay. The data of each study were tested thrice. *p < 0.05, **p < 0.01, ***p < 0.001.

We found that the expression and acetylation level of FOXA2 in OGD/R-induced HT22 cells were abnormally upregulated, whereas FOXA2 expression was downregulated and the FOXA2 acetylation level was decreased after baicalein treatment (Figs. 4*A–C*). SIRT6 regulates deacetylation of downstream proteins and participates in disease progression (Chang et al., 2020). Combined with previous results showing that baicalein upregulates SIRT6 expression, we speculated that baicalein regulates FOXA2 deacetylation through SIRT6. To further explore whether SIRT6 affects FOXA2 acetylation, HT22 cells were treated with an HDAC family inhibitor (TSA) and a sirtuin family inhibitor (NAM). The results revealed that NAM upregulated FOXA2 acetylation and protein expression, whereas TSA had little effect on FOXA2 acetylation and protein expression (Fig. 4*D*). Additionally, sh-SIRT6 upregulated FOXA2 acetylation and protein expression, whereas pcDNA3.1-SIRT6 inhibited FOXA2 acetylation and protein expression (Fig. 4*E*). Co-IP assays confirmed the interaction between SIRT6 and FOXA2. Moreover, the interaction between SIRT6 and FOXA2 was attenuated in the OGD/R-induced HT22 cells. Remarkably, baicalein enhanced the interaction between SIRT6 and FOXA2 in the OGD/R-induced HT22 cells (Fig. 4*F*). Furthermore, SIRT6 and FOXA2 were colocalized in the nucleus of HT22 cells (Fig. 4*G*). Finally, sh-SIRT6 enhanced FOXA2 acetylation level and protein expression in OGD/R-induced HT22 cells, reversing the effects of baicalein on FOXA2 acetylation and protein expression (Fig. 4*H*). These results suggested that baicalein promoted FOXA2 deacetylation by upregulating SIRT6 expression.

### SIRT6 inhibited the transcriptional regulation of SLC7A11 by FOXA2

**Figure 5.**
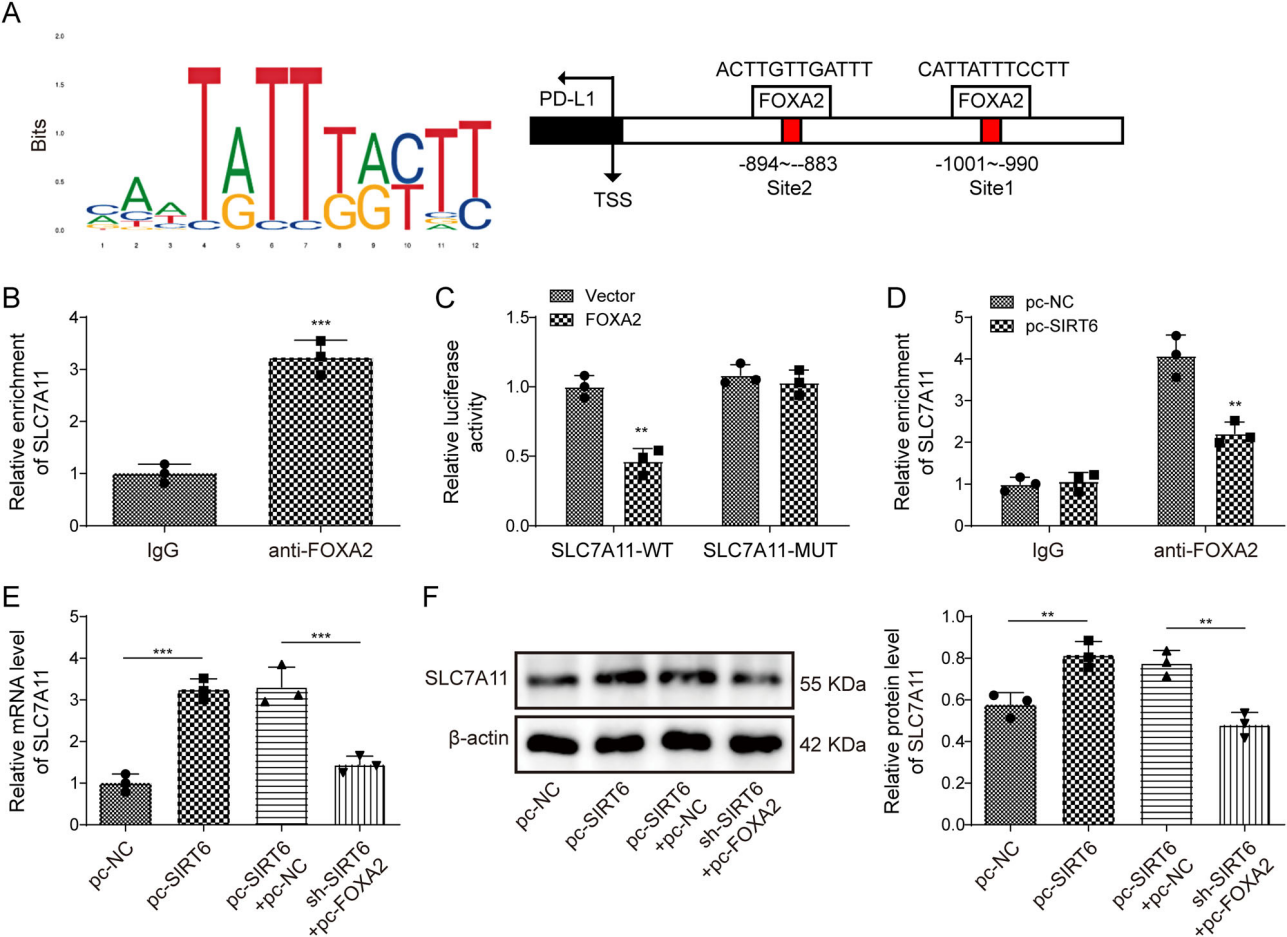
SIRT6 inhibited the transcriptional regulation of SLC7A11 by FOXA2. OGD/R-induced HT22 cells were treated with baicalein and transfected with the pcDNA3.1-SIRT6. ***A***, The potential binding site of FOXA2 on SLC7A11 was analyzed using the StarBase database. ***B–D***, Dual-luciferase reporter and ChIP assays were used to substantiate the relationship between FOXA2 and SLC7A11. ***E***, ***F***, SIRT6 mRNA and protein expression were substantiated by qRT-PCR and Western blot analysis (WB). Data from each study were tested thrice. *p < 0.05, **p < 0.01, ***p < 0.001.

Subsequently, we explored the downstream mechanisms of FOXA2. JASPAR database predicted the presence of a binding site between FOXA2 and SLC7A11 (Fig. 5*A*). The ChIP assay revealed that anti-FOXA2 enriched SLC7A11 to a greater extent than IgG (Fig. 5*B*). The dual-luciferase reporter gene assay confirmed that FOXA2 effectively inhibited the luciferase activity of SLC7A11-WT but had a marginal effect on the luciferase activity of SLC7A11-MUT (Fig. 5*C*). SIRT6 overexpression attenuated the interaction between FOXA2 and SLC7A11 (Fig. 5*D*). Additionally, SIRT6 overexpression upregulated the expression of SLC7A11 in OGD/R-induced HT22 cells, which was reversed by FOXA2 overexpression (Fig. 5*E*,*F*). These results imply that SIRT6 inhibited the transcriptional regulation of SLC7A11 by FOXA2.

### SIRT6 regulated OGD/R-induced HT22 cell partial role of ferroptosis through FOXA2

**Figure 6.**
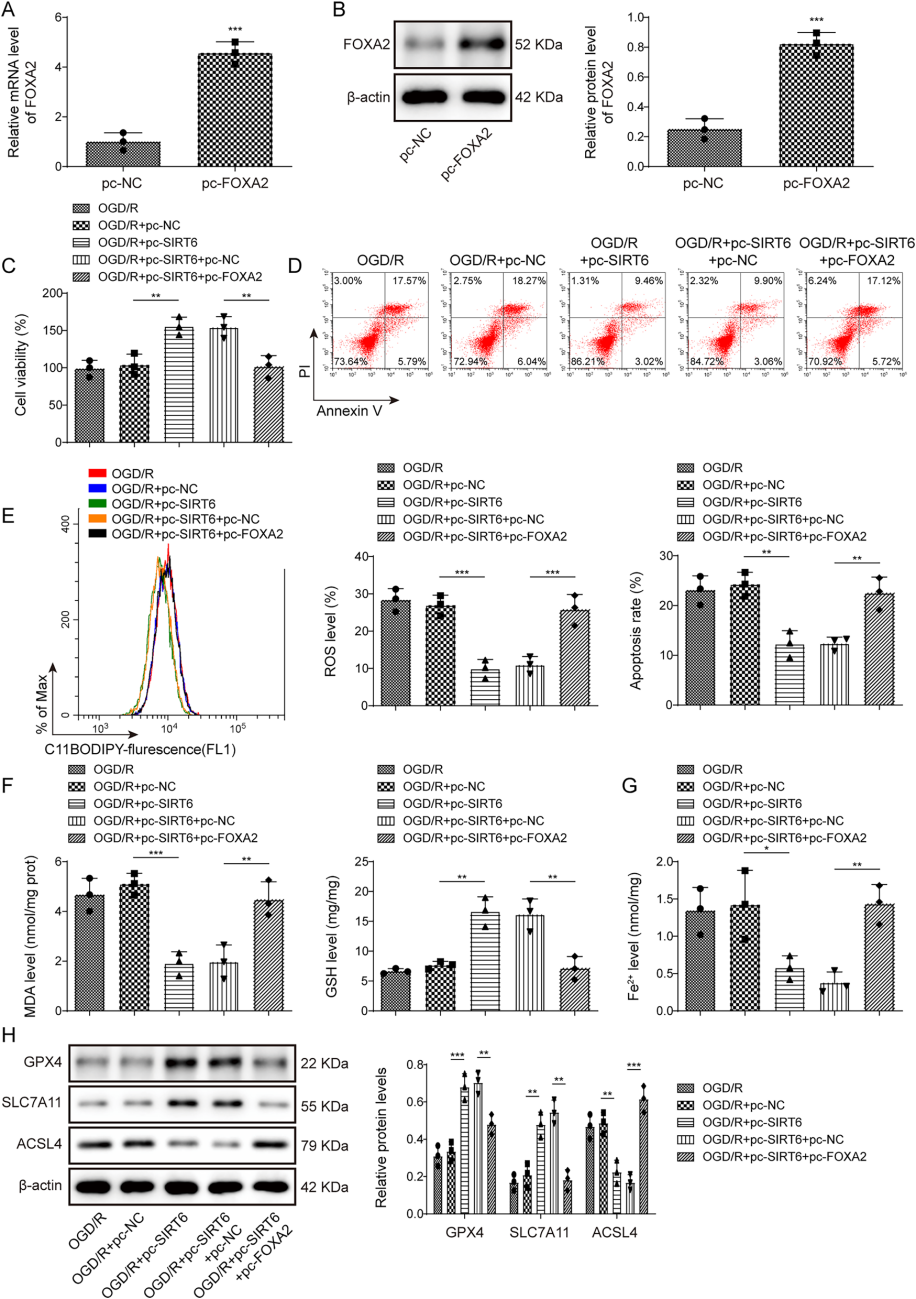
SIRT6 regulated OGD/R-induced HT22 cell partial role of ferroptosis through FOXA2. OGD/R-induced HT22 cells were treated with baicalein and transfected with pcDNA3.1-FOXA2 or pcDNA3.1-SIRT6. ***A***, ***B***, FOXA2 mRNA and protein expression were substantiated by qRT-PCR and WB. ***C***, Cell viability was determined using the CCK-8 assay. ***D***, The cell death rate was ascertained using flow cytometry. ***E–G***, Lipid ROS, MDA, GSH, and Fe^2+^ levels were measured using kits. ***H***, Expression of GXP4, SLC7A11, and ACSL4 was determined by Western blotting. Data from each study were tested thrice. *p < 0.05, **p < 0.01, ***p < 0.001.

To investigate the role of SIRT6/FOXA2 in I/R cell models, HT22 cells were transfected with pcDNA3.1-FOXA2, which resulted in the upregulation of FOXA2 mRNA and protein expression (Fig. 6*A*,*B*). SIRT6 upregulation increased the viability of OGD/R-induced HT22 cells and decreased the cell death rate, whereas FOXA2 overexpression attenuated SIRT6 upregulation (Fig. 6*C*,*D*). In addition, in OGD/R-induced HT22 cells overexpressing SIRT6, lipid ROS and MDA levels were inhibited, and GSH levels were increased (Fig. 6*E*,*F*). Moreover, Fe^2+^ levels were decreased, GPX4 and SLC7A11 protein expression was upregulated, and ACSL4 protein expression was inhibited after SIRT6 overexpression (Fig. 6*G*,*H*). Nevertheless, pcDNA3.1-FOXA2 effectively reversed the effects of pcDNA3.1-SIRT6; enhanced the levels of ROS, MDA, and Fe^2+^; downregulated GSH levels; inhibited GPX4 and SLC7A11 expression; and promoted ACSL4 protein expression (Fig. 6*G*,*H*). These results revealed that SIRT6 partially regulated ferroptosis in OGD/R-induced HT22 cells through FOXA2.

### Baicalein inhibited partial role of ferroptosis by upregulating SIRT6 to alleviate I/R injury in mice

**Figure 7.**
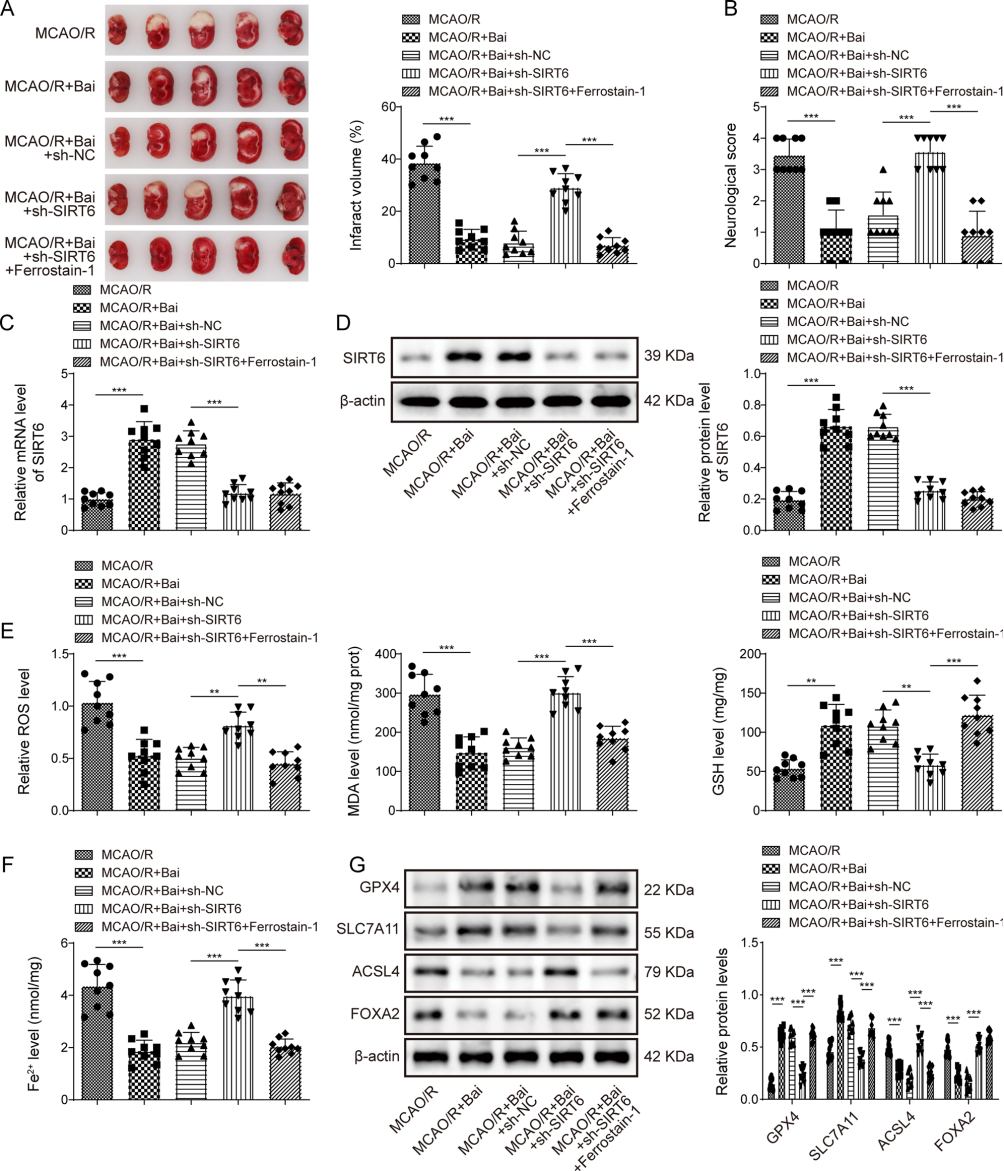
Baicalein inhibited partial role of ferroptosis by upregulating SIRT6 to alleviate I/R injury in mice. The MCAO/R mice were injected with baicalein and sh-SIRT6. ***A***, The infarct size was measured using TTC staining. ***B***, Neurological deficit scores were used to evaluate the nerve injuries. ***C***, ***D***, SIRT6 mRNA and protein expression were substantiated by qRT-PCR and Western blotting. ***E***, ***F***, The levels of MDA, GSH, Fe^2+^ were detected using kits. ***G***, The expression of GXP4, SLC7A11, ACSL4, and FOXA2 was determined by Western blotting. *n* = 9. *p < 0.05, **p < 0.01, ***p < 0.001.

Finally, we confirmed that baicalein ameliorates cerebral I/R injury in mice by regulating SIRT6 expression to inhibit partial role of ferroptosis. In the brain tissues of SIRT6 knockdown mice, the infarct size and neurological deficit score increased, which attenuated the therapeutic effect of baicalein (Fig. 7*A*,*B*). However, Ferrostain-1 reversed the function of SIRT6 knockdown and decreased the infarct size and nerve damage score in the mice brain tissue (Fig. 7*A*,*B*). Although baicalein upregulated SIRT6 expression in MCAO/R mice brain tissue, sh-SIRT6 inhibited SIRT6 expression in baicalein-treated MCAO/R mice brain tissue. Interestingly, Ferrostain-1 had no effect on the expression of SIRT6 (Fig. 7*C*,*D*). In addition, sh-SIRT6 effectively attenuated the protective effects of baicalein. In the brain tissue of baicalein-treated MCAO/R mice, SIRT6 knockdown increased the levels of MDA and Fe^2+^, decreased the levels of GSH, downregulated GPX4 and SLC7A11 expression, and upregulated ACSL4 and FOXA2 expression (Fig. 7*E–G*). It was worth noting that Ferrostain-1 effectively attenuated the effect of sh-SIRT6 in baicalein-treated MCAO/R mice brain tissue (Fig. 7*E–G*). These results confirmed that baicalein partially inhibited ferroptosis by upregulating SIRT6, thereby alleviating cerebral I/R injury in mice.

## Discussion

The recovery of blood flow after cerebral ischemia may lead to tissue injury aggravation and energy metabolism disturbance of neurons in the penumbra after reperfusion, which involves various mechanisms such as inflammatory activation, oxidative stress, and ferroptosis (Y. Chen et al., 2021d). This study demonstrates that baicalein alleviates I/R injury in MCAO/R mice brain tissue and OGD/R-induced HT22 cells by inhibiting apoptosis and partial role of ferroptosis.

Previous studies have shown that baicalein inhibits cellular oxidative stress injury and decreases the volume of cerebral ischemia, thus ameliorating motor dysfunction in patients with IS (Tang et al., 2023). Additionally, baicalein regulates FTH1 to reduce intracellular total iron and Fe^2+^ levels (M. Yang et al., 2021). Li et al. confirmed that baicalein inhibited ferroptosis and ameliorated cerebral I/R injury by regulating GPX4/ACSL4/ACSL3 axis (M. Li et al., 2022). In this study, using animal and cellular models, we found that baicalein inhibited ferroptosis and reduced oxidative stress, thus alleviating I/R injury. Subsequently, the downstream mechanisms of baicalein were explored. SIRT6 is overexpressed in the brain (He et al., 2021). Recently, SIRT6 could have a protective function against I/R injury. SIRT6 expression was decreased in MCAO rat model and OGD/R-induced human neuroblastoma cell (SHSY5Y; Lee et al., 2013). SIRT6 overexpression plays a protective role in mice cerebral I/R model and OGD/R-stimulated mice neuro-2a neuroblastoma cells (N2a cells; Zhang et al., 2017). Similar to previous studies, we found that SIRT6 expression was abnormally inhibited in MCAO/R mice brain tissue and OGD/R-induced HT22 cells. Notably, baicalein effectively upregulated the expression of SIRT6. Moreover, in I/R animal and cell models, we found that sh-SIRT6 attenuated the effect of baicalein on cerebral ischemic injury and its inhibitory effect on ferroptosis. Interestingly, Ferrostain-1 effectively attenuated the function of sh-SIRT6 without affecting the expression of SIRT6, thereby restoring the therapeutic effects of baicalein. This evidence indicates that baicalein upregulates SIRT6, thereby inhibiting ferroptosis and alleviating I/R injury, further emphasizing that SIRT6 is the target of baicalein, which is expected to be a potential therapeutic target for IS. Ferroptosis is closely related to apoptosis and can be transformed into ferroptosis under certain conditions, which increases the sensitivity of cells to apoptosis (Yi et al., 2020). Furthermore, baicalein induces ferroptosis and apoptosis in multiple cancers and plays an anticancer role (Kong et al., 2021; J. Li et al., 2024; Lai et al., 2024). However, baicalein ameliorates cerebral I/R injury by inhibiting neuronal ferroptosis (M. Li et al., 2022). Additionally, baicalein can inhibit the apoptosis of OGD cells, relieve oxidative stress, protect mitochondrial function, and restore mitochondrial membrane potential, thereby alleviating cerebral I/R injury (W. H. Li et al., 2020). Yang et al. revealed that baicalein alleviated subacute cerebral I/R injury by alleviating neuroinflammation, apoptosis, and autophagy and played a neuroprotective role (S. Yang et al., 2019). In our study, the expression of proapoptotic proteins was upregulated, and the expression of antiapoptotic proteins was downregulated in MCAO/R mice, whereas the improvement trend was ameliorated after treatment with baicalein. This finding enriches the study of baicalein-induced apoptosis to ameliorate IS.

Finally, the mechanisms downstream of SIRT6 were explored. FOXA2-mediated metabolic transcription is controlled by SIRT1 (van Gent et al., 2014). In this study, we found that SIRT6, which belongs to the same family as SIRT1, interacts with the FOXA2 protein and that SIRT6 overexpression inhibits the expression and acetylation of FOXA2. There are few reports on the function of FOXA2 in IS. The expression and acetylation of FOXA2 were abnormally upregulated in OGD/R-induced HT22 cells. Furthermore, FOXA2 overexpression intensified oxidative stress and facilitated ferroptosis. FOXA2 transcription has been reported to inhibit target gene expression, thus aggravating I/R-injured heart (Gao et al., 2022). Importantly, FOXA2 transcription negatively regulates SLC7A11 expression. The inhibition of SLC7A11 expression can induce ferroptosis (Lei et al., 2021). Yuan et al. reported that kaempferol enhanced the protective effect on OGD/R-induced cell ferroptosis by increasing SLC7A11 (Yuan et al., 2021). Galangin upregulated SLC7A11 expression and inhibited the expression of ferroptosis markers (W. Chen et al., 2021a). Consistent with this, we found that SLC7A11 expression was downregulated in IS but upregulated after baicalein treatment. Nevertheless, the roles of FOXA2 in MCAO/R and baicalein in cerebral I/R injury require further exploration. Additionally, this study was conducted using only cellular and animal models. If future conditions permit, relevant clinical samples should be collected to further explore the roles and mechanisms of SIRT6 and FOXA2 in cerebral I/R injury. Simultaneously, considering that baicalein can inhibit ferroptosis and ameliorate brain injury, whether there is a synergistic effect between baicalein and ferroptosis inhibitors is also worth further investigation. Baicalein, the primary active flavonoid in *Scutellaria baicalensis*, plays a neuroprotective role by reducing neuroinflammation, inhibiting ferroptosis and apoptosis, and alleviating cerebral I/R injury (S. Yang et al., 2019; M. Li et al., 2022). At present, the quantity and quality of clinical studies on baicalein in the treatment of IS are limited, and further clinical trials with large sample sizes and long-term follow-up are needed to verify its efficacy and safety. Considering that the dosage of baicalein is affected by specific diseases, individual differences, and drug compatibility, further studies are needed to improve the baicalein dosage form and route of administration to better meet clinical needs.

**Figure 8.**
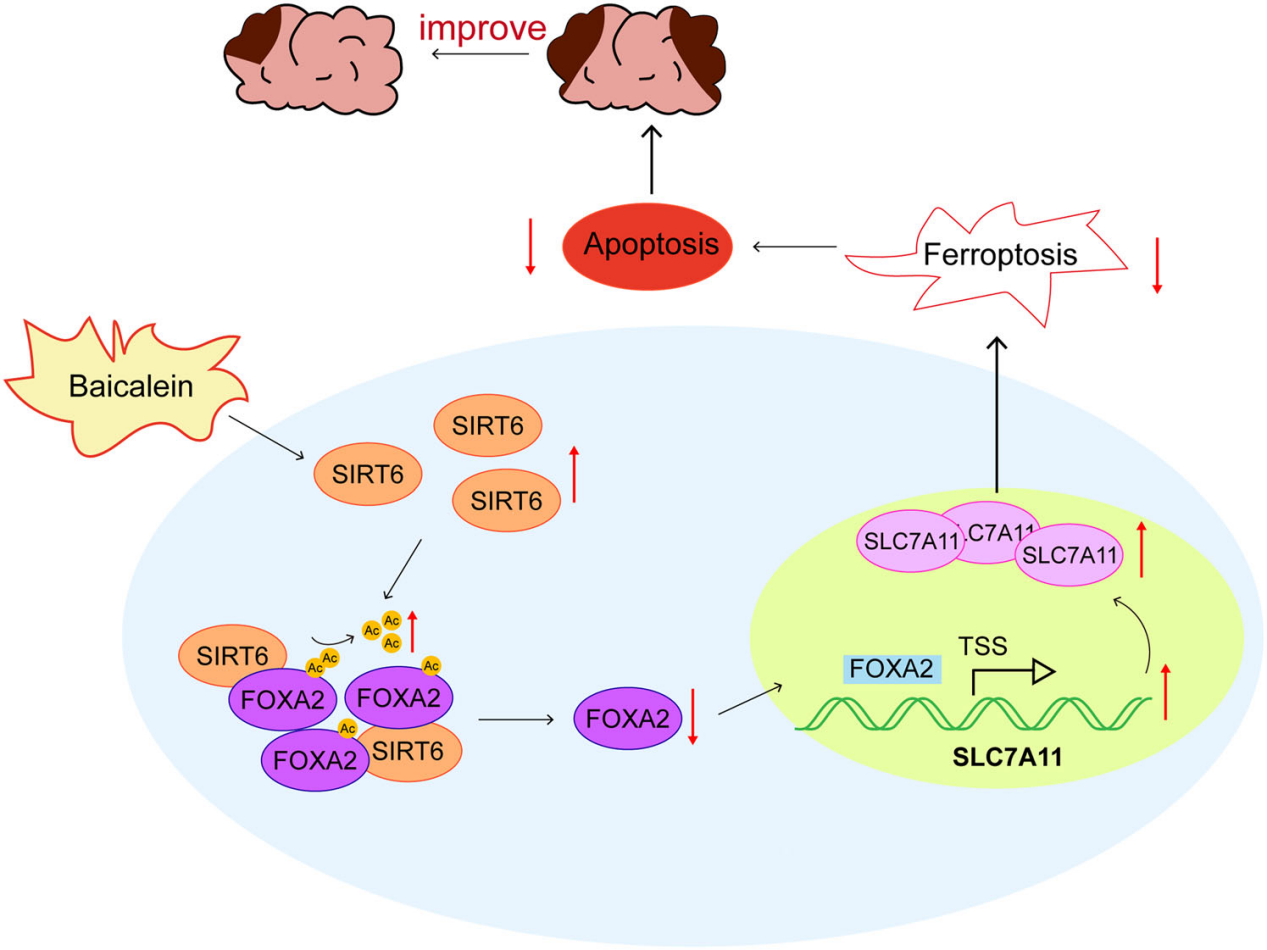
Baicalein promotes FOXA2 deacetylation by upregulating SIRT6 expression, thereby inhibiting FOXA2 transcription, leading to the upregulation of SLC7A11 expression, inhibiting ferroptosis, inhibiting cell apoptosis, and ultimately alleviating I/R injury in IS.

In summary, this study confirmed, for the first time, that baicalein promotes FOXA2 deacetylation by upregulating SIRT6 expression, thereby inhibiting FOXA2 transcription, leading to the upregulation of SLC7A11 expression, inhibiting ferroptosis, inhibiting cell apoptosis, and ultimately alleviating I/R injury in IS (Fig. 8). Thus, this study identified a novel compound for IS therapy.

## Synthesis

Reviewing Editor: Eran Perlson, Tel Aviv University Decisions are customarily a result of the Reviewing Editor and the peer reviewers coming together and discussing their recommendations until a consensus is reached. When revisions are invited, a fact-based synthesis statement explaining their decision and outlining what is needed to prepare a revision will be listed below. The following reviewer(s) agreed to reveal their identity: NONE.

Thank you for the opportunity to review the manuscript entitled "Baicalein inhibits cerebral ischemia-reperfusion injury ferroptosis through SIRT6-mediated FOXA2 deacetylation to promote SLC7A11 expression." The study is interesting, novel, and important with potential clinical application. The authors demonstrate that baicalein treatment protects brain tissue against ischemia-reperfusion (IR) injury, improving neurological scores compared to controls.

The research advances several areas, including:

1. Mechanism of Ischemic Stroke (IS): The study investigates the role of ferroptosis in ischemic stroke, revealing its crucial contribution to ischemia-reperfusion injury. Inhibiting ferroptosis can effectively alleviate IR injury, providing new targets for ischemic stroke treatment.

2. Regulation of Ferroptosis: The article explores expression changes in ferroptosis-related proteins (GPX4 and SLC7A11) and their impact on IR injury. It details how SIRT6 and FOXA2 regulate ferroptosis, with SIRT6 inhibiting FOXA2 expression and acetylation, leading to upregulation of SLC7A11 and suppression of ferroptosis.

3. Role of Baicalein in Neuroprotection: The research shows that baicalein alleviates cerebral ischemia-reperfusion injury by upregulating SIRT6 expression, promoting FOXA2 deacetylation, and ultimately inhibiting ferroptosis. This provides scientific evidence for baicalein as a potential therapeutic agent for ischemic stroke.

4. Neuroprotection Mechanism Research: By establishing stroke mouse models (MCAO/R) and cell models (OGD/R-induced HT22 cells), the study investigates baicalein's specific mechanisms in neuroprotection, laying a theoretical foundation for developing new neuroprotective agents.

Notably, baicalein administration after the occlusion phase and before reperfusion aligns with potential clinical applications during thrombolytic therapy with tPA for ischemic stroke. The role of the SIRT6-FOXA2-SLC7A11 pathway is supported by extensive in vivo and in vitro data. However, to strengthen the generalizability of these findings, the study could benefit from exploring alternative mechanisms alongside the proposed anti-ferroptosis effect. For instance, baicalein's influence on non-ferroptosis cell death pathways, such as the intrinsic apoptotic pathway, remains unclear. Previous research suggests baicalein's pro-apoptosis role in non-neuronal cells (PMID: 33585556). Evaluating apoptotic markers like Bcl-2 family protein levels, cytochrome c release, and caspase activation in the brain could address this gap.

Further suggestions for Improvement:

1. Submission of Original Images: While the visual abstract is informative and accurately conveys scientific information, the original Western blot images are missing. Including these images is essential for verifying the results and ensuring transparency. Please submit the original Western blot images.

2. Discussion of Limitations: Include a section discussing the limitations of your study. Address potential confounding factors, limitations in experimental design, and any challenges faced during the study. This transparency will strengthen your conclusions and provide a balanced view of your research.

3. Future Directions: Outline future research directions based on your findings. Suggest how your research can be expanded or applied in clinical settings. This will provide a clear path for subsequent studies and applications.

4. Clarification of Treatment Course: The treatment course with baicalein and other treatments needs to be clearly highlighted. For example, on page 5, line 73: "..., the treatment time was 1, 4, and 7 days, respectively," but it is not clear concerning what point. Adding a timeline in figure 1 would be very helpful.

5. Editing for Clarity and Precision: There are points across the text that require editing. Some examples include:

o Page 8, line 136: "cells ... were vaccinated ..."

o Page 8, line 137: "cells were collected and laundered."

o Page 5, line 85: "Ethics committee of XXX"

o Page 4, line 60-61: Clarify the sentence "It is important that there are potential binding sites between FOXA2 and SLC7A11" to "... potential FOXA2 binding sites near the SLC7A11 promoter."

o Page 4, line 64: Revise the tone of "this study aimed to confirm ..." to reflect that studies usually aim to explore possibilities, not confirm original hypotheses.

o Page 13, line 224: To avoid confusion, suggest changing "the apoptosis rate decreased ..." to "...cell death rate decreased ...".

## Author Response

Dear Editor, Thank you for your letter and comment our manuscript titled "Baicalein inhibits cerebral ischemia-reperfusion injury through SIRT6-mediated FOXA2 deacetylation to promote SLC7A11 expression". These comments helped us improve our manuscript, and provided important guidance for future research. We have addressed the editor's and the reviewers' comments to the best of our abilities, and revised text to suitable for publication in eNeuro.

Sincerely yours.

Your revision must include the manuscript with new text indicated in a bold or highlighted font to aid the re-review of the manuscript. Separately, please provide a clean copy of the manuscript that includes the title page, which will be published if accepted. Please closely review your manuscript at this time for any final corrections in style or substance. Please consult our Revised Submission Checklist for full details on preparing your revision: https://www.eneuro.org/content/revising-finalizing-manuscript#revisions Response: Thank you very much for your comments. According to the suggestion, we have highlighted the revised content in the revised manuscript and submitted a clean copy of the manuscript that included the title page in order to meet the requirements for publication in your journal. Please refer to the revised manuscript and figures for details.

In an effort to improve rigor, transparency, and reproducibility, eNeuro strongly encourages the use of estimation statistics. If the use of estimation statistics applies to your research, you may consider including it in your revision. More details and resources can be found in the Information for Authors: https://www.eneuro.org/content/preparing-manuscript#statistics Response: Thank you very much for your comments. We apologize that our data are not suitable for estimation statistics. For instance: 1. most of our data consisted of data from three or more groups (five group) (figure 3\4\6\7), which should be analyzed using Cumming estimation plot (as shown in the following figure) according to the suggestion. However, the link of Cumming estimation plot cannot be opened. 2. We did our best to evaluate Figures 1 and 2 (four groups) using Multi two-group estimation plot. It was found that Figures 1B-C (five groups) lost the results of day 7 group (as shown in the following figure), which is also confirmed that odd-numbered groups are not suitable for the Multi two-group estimation plot. Moreover, we have provided the figures and data of estimation statistics in Figures 1 and 2 (four groups) as shown in the following figure. We found that most of the results were not statistically significant of the following figure using estimation statistics. Therefore, the following figures were not cited in the manuscript, which was only quoted in point-by-point reply. Moreover, we have revised the picture presentation as far as possible according to the requirements, such as in the form of scatter charts and column charts. Please refer to the revised manuscript and figures for details. The above is our understanding of this problem, and we hope that our revision can meet the requirements. If there are any questions later, please do not hesitate to contact us.

Your submission must include publication-quality figures, each in a separate EPS or TIFF (300 dpi) file. Please make sure your figures adhere to style requirements to avoid delays in manuscript processing. Detailed guidelines for figures are available in our Information for Authors: https://www.eneuro.org/content/preparing-manuscript#figures Response: Thank you very much for your comments. We provided TIFF (300 dpi) pictures and labeled the molecular weight of the protein. To facilitate the understanding of red-green colorblind readers, we changed the color of the bar chart to different textures or lines for display. Please refer to the revised manuscript and figures for details.

Synthesis of Reviews:

Computational Neuroscience Model Code Accessibility Comments for Author (Required): a Synthesis Statement for Author (Required):

The revised manuscript of "Baicalein inhibits cerebral ischemia-reperfusion injury ferroptosis through SIRT6-mediated FOXA2 deacetylation to promote SLC7A11 expression" have been significantly improved in this revision. This includes adding the timeline (fig 1A), individual data points in all the histograms, additional data, and the extended discussion. The manuscript provides evidence to support basis for a potential new treatment for re-perfusion injury after ischemic stroke. It presents a complex and scientifically significant topic with clarity and precision. The research approach seems highly rigorous and the findings have the potential to make a significant contribution to the field of cerebral ischemia-reperfusion injury. The exploration of the mechanism through SIRT6-mediated FOXA2 deacetylation and its impact on SLC7A11 expression is both innovative and insightful.

The added data on classical intrinsic apoptosis pathway (Bax, Bcl-2, Cyt-c, and caspase-3) clearly show that baicalein acts as an anti-apoptotic factor in this paradigm. Therefore, the potential therapeutic role of baicalein by inhibiting ferroptosis after I/R injury is partial. This fact should be reflected in the tone across the manuscript. This finding shed light on the possible crosstalk between the two pathways has been well-explained in the discussion section and perhaps should be explored in future works. However, it is important to be careful and balance the role of these pathways in the rest of the text. For example, the sentence, "These results suggested that baicalein could alleviate I/R nerve injury in mice brain tissed by inhibiting ferroptosis, and this mechanism might be achieved by upregulating SIRT6 expression" (lines 239-41) needs to be revised not only for grammatical and spelling errors but also to reflect the partial role of ferroptosis in this paradigm. This should also be reflected in the title of the manuscript.

Response: Thank you very much for your comments. According to the suggestion, we revised the above sentence to: "...... alleviate nerve injury in MCAO/R mice brain tissue by inhibiting the partial role of ferroptosis, and this mechanism ......". In addition, "ferroptosis" was removed from the title of the manuscript, and the resulting description of ferroptosis also indicated partial role of ferroptosis, and apoptosis was added in the result of Figure 1. Please refer to the revised manuscript and figures for details.

Minor:

1- Text still needs to be thoroughly checked for usage and occasional spelling errors. For example, it is unusual to use the word "launder" instead of "wash" in the context of sample preparation from cultured cells (line 152). Other examples include but not limited to:

- "... threatens the life quality of patients" (Line 8) - " ... to regulate IS." (line 10) - " ... alleviate I/R nerve injury in mice brain tissed .." (line 240) Response: Thank you very much for your comments. According to the suggestion, "launder" was revised to "washed", "... threatens the quality of life of patients" was revised to "... poses a serious threat to patient survival", "... to regulate IS." was revised to "... for the treatment of ischemic stroke", "... alleviate I/R nerve injury in mice brain tissed .." was revised to "... alleviate nerve injury in MCAO/R mice brain tissue by inhibiting apoptosis and the partial role of ferroptosis...". Additionally, the full paper was carefully checked for grammar and word spelling errors, while native English speakers were invited to revise the manuscript. In addition, the manuscript was sent to the professional structure for polishing and the proof of polishing was provided. Please refer to the revised manuscript and figures for details.

2- Fig 3E and 6E: Removing the top horizontal and right vertical lines of the graphs helps with consistent appearance of figures and avoid interrupting the graph legends.

Response: Thank you very much for your comments. According to the suggestion, we adjusted Fig. 2C, 3E and 6E, so that their groups can be clearly displayed. Please refer to the revised manuscript and figures for details.
